# Temporal Evolution and Stationary Point Mechanisms of Pelvic Characteristic Angles Throughout the Sit‐to‐Stand Cycle

**DOI:** 10.1111/os.70366

**Published:** 2026-07-08

**Authors:** Houwen Zheng, Chen‐Xu Liu, Haoyue Xin, Hua Tian, Zhanli Liu, Minwei Zhao

**Affiliations:** ^1^ Department of Engineering Mechanics, Applied Mechanics Lab, School of Aerospace Tsinghua University Beijing China; ^2^ Department of Mechanics Beijing University of Technology Beijing China; ^3^ Department of Orthopaedics Peking University Third Hospital Beijing China; ^4^ Engineering Research Center of Bone and Joint Precision Medicine, Ministry of Education Beijing China

**Keywords:** kinematics, pelvis, postural transitions, total hip arthroplasty

## Abstract

**Objectives:**

Prosthetic impingement and dislocation remain prevalent complications following total hip arthroplasty (THA). The “safe zone” concept guiding acetabular component placement is predicated on static body positions, while neglecting the kinematic changes in pelvic angles throughout the stand‐to‐sit (STS) transition cycle. This static‐centric approach constitutes a primary contributing factor to THA‐related complications. Consequently, investigating the kinematic regularity of pelvic motion across the entire STS cycle is of particular significance for optimizing surgical strategies and improving postoperative outcomes.

**Methods:**

Dynamic pelvic angles are analyzed in 10 healthy individuals performing STS using optical motion capture combined with biodynamics simulations. The skeletal model is scaled to each subject, and inverse kinematics is used to compute motion trajectories, from which the time‐varying characteristics of sacral slope (SS), pelvic incidence (PI), and pelvic tilt (PT) angles are extracted.

**Results:**

The skeletal model of the STS movement cycle is simulated, revealing the nonlinear variations in pelvic characteristic angles. Notably, the variation curves of SS and PT angles both exhibit distinct stationary points during the critical phase of the movement cycle, with the two parameters showing opposite changing trends. Specifically, PT angle decreases sharply to a trough within the 44.48%–53.19% interval of the movement cycle and then gradually rebounds from this stationary point. In contrast, SS angle increases first and then decreases, with its stationary point highly coinciding with that of PT. At the trough stationary point of PT or SS, body center of gravity (COG) trajectory exhibits a strong correlation with PT or SS kinematics and synchronous transitions in displacement velocity and direction, highlighting the crucial role of pelvic posture adjustment in body COG regulation.

**Conclusions:**

A biomechanical framework for investigating the kinematic characteristics of pelvic angles during the STS task is proposed. In healthy individuals, the kinematic patterns of pelvic motion during STS demonstrate prominent nonlinear characteristics, accompanied by distinct stationary points in the pelvic characteristic angles around the mid‐STS phase. The current acetabular safe zone is insufficient to account for dynamic variations in pelvic orientation, and the identified critical stationary points of pelvic angles during the mid‐STS phase may provide theoretical support for preoperative planning and postoperative rehabilitation in patients undergoing THA.

## Graphical abstract

Dynamic motion‐capture and musculoskeletal simulation of stand‐to‐sit reveal nonlinear pelvic kinematics in healthy subjects. Pelvic tilt and sacral slope show synchronized mid‐cycle turning points, strongly linked to center‐of‐gravity transitions. These dynamic features, absent in static radiographs, may refine functional acetabular safe zones to reduce THA impingement/dislocation.

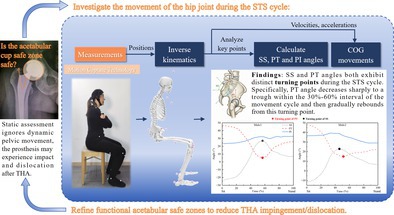

## 1 Introduction

Total hip arthroplasty (THA) has become the gold standard for treating end‐stage hip osteoarthritis and other hip disorders, effectively relieving pain and restoring hip function in millions of patients worldwide [1, 2]. The long‐term success of THA is highly dependent on the precise placement of the acetabular component, as suboptimal positioning is closely associated with increased risks of postoperative complications, including prosthetic impingement, dislocation, and limited range of motion (ROM) [3, 4]. To mitigate these risks, the concept of an “acetabular safe zone” has been widely accepted, defining an optimal range of acetabular abduction angle and anteversion angle based on static anteroposterior (AP) radiographs of the pelvis in the standing position [5, 6].

Despite the widespread adoption of this static safe zone, clinical practice still faces significant challenges. A considerable proportion of patients experience residual hip discomfort or functional limitations after THA, even when acetabular components are placed within the predefined static safe zone [7, 8]. Recent clinical studies have reported that up to 10%–15% of THA patients suffer from prosthetic impingement or instability during daily activities, leading to reduced quality of life and increased revision rates [9, 10]. These findings suggest that the static safe zone, which only reflects pelvic and prosthetic positions in a single upright posture, may fail to capture the dynamic changes in pelvic alignment during functional movements.

Human hip joint function is inherently dynamic, and daily activities such as sitting, standing, and walking involve complex pelvic motions (e.g., tilting, rotation, and translation) [11, 12]. The sit‐to‐stand (STS) movement, one of the most frequent and essential functional activities in daily life, requires significant pelvic mobility and hip joint excursion [13]. During STS, the pelvis undergoes substantial posterior tilting and rotational changes, which can alter the relative position between the acetabular component and the femoral head, potentially pushing the prosthesis beyond the statically defined safe zone even if it was optimally positioned in the standing state [14, 15]. However, current research on acetabular component positioning primarily relies on static imaging evaluations, and there is a critical lack of systematic studies investigating the dynamic changes in pelvic characteristic angles during functional movements.

Given the limitations of static assessments and the clinical significance of dynamic hip joint function, investigations into the dynamic variation patterns of the spino‐pelvic complex have been conducted by several researchers. For healthy individuals, standardized STS descriptions have been established, defining six core events (e.g., seat unloading initiation, seat‐off, momentum transfer termination) and five phases, with kinematics/kinetics characterized via force plates and motion capture [16, 17]. Clinical studies focus on STS in patient populations: adults with spinal deformity (ASD) show reduced pelvic anteversion/hip flexion in sagittal malalignment subgroups, greater thoracic kyphosis mobility in turning subgroups [18]; stroke patients with spastic hemiplegia exhibit elevated hip/knee joint reaction forces positively correlated with joint moments and weight‐bearing asymmetry [13]; chronic low back pain individuals display greater lumbar motion ranges during STS versus healthy controls; and lower limb prosthesis users use upper limb assistance to complete STS, with performance linked to amputation level, etiology, and functional classification [19]. However, prior studies mainly focus on isolated joint angles/moments or global parameters (e.g., movement time, joint reaction forces) rather than the integrated spino‐pelvic complex, hindering comprehensive understanding of STS movement mechanisms given the pivotal role of spino‐pelvic coordination in movement stability, load distribution, and functional efficiency [20, 21].

In this study, we focus on quantifying pelvic kinematics during the STS transition in healthy individuals. A 3D simulation framework is established based on optical motion capture and OpenSim, enabling the extraction of time‐varying pelvic characteristic angles over the entire STS cycle. Sacral slope (SS), pelvic tilt (PT), and pelvic incidence (PI) are selected as core parameters for their critical role in spino‐pelvic biomechanics. Particular attention is given to identifying key postural stationary points in these angles and determining their temporal relationship with the trajectory of the body's center of gravity (COG). By characterizing these dynamic patterns and critical stationary points, this work aims to provide a methodological and biomechanical basis for future dynamic optimization of the acetabular safe zone in THA patients.

## 2 Methods

**FIGURE 1:**
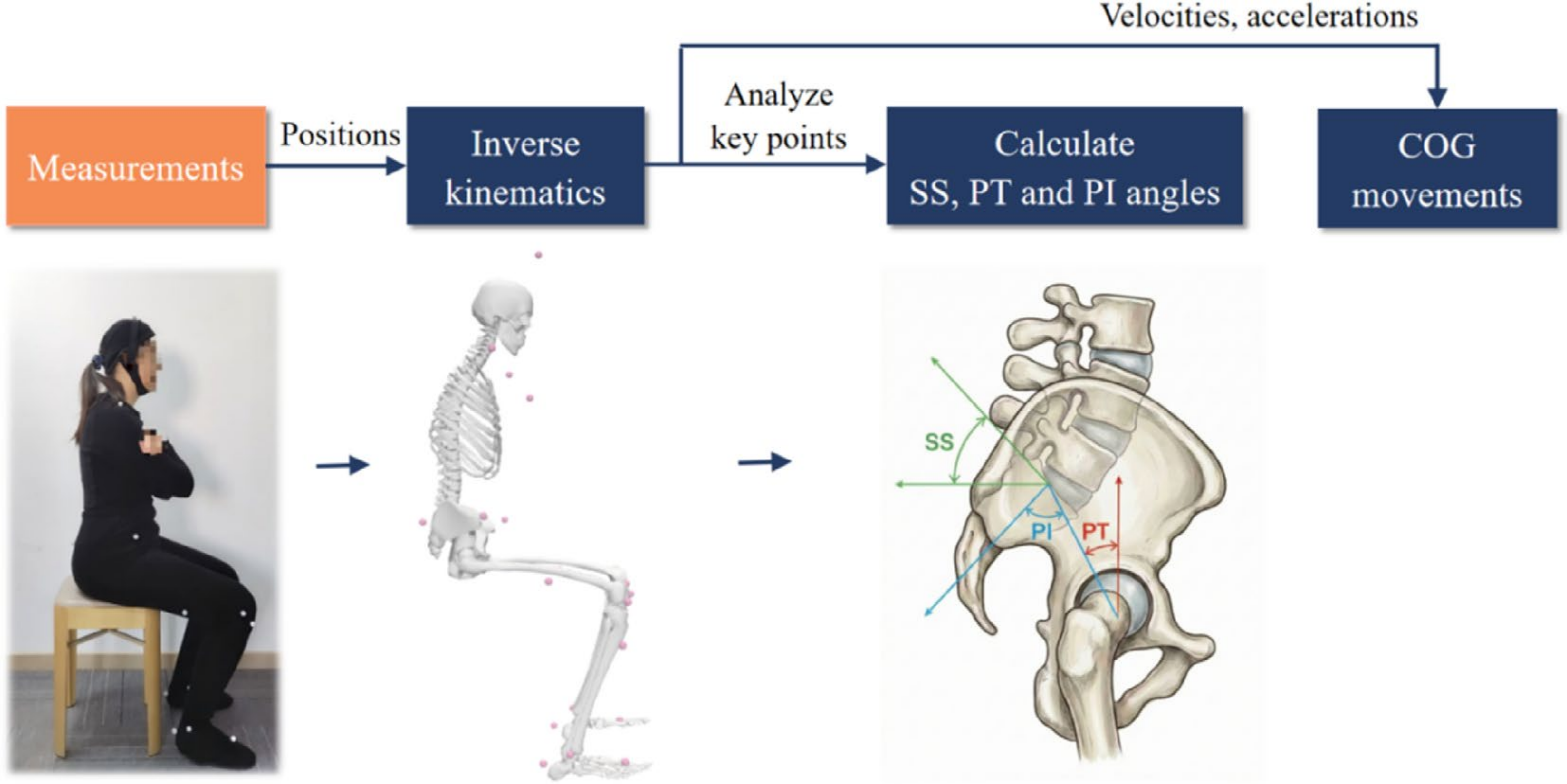
Backward simulation progress. First, motion data of STS are acquired via motion capture experiments. Then, inverse kinematics analysis is performed on the collected data. Key points are extracted to calculate the dynamic variation patterns of pelvic characteristic angles. Concurrently, the movement characteristics of the center of gravity (COG) are analyzed alongside these angle data.

This study focuses on SS, PT and PI (PI = SS + PT) because these parameters are critical to understanding lumbar‐pelvic biomechanics: PI determines lumbar‐pelvic stiffness, compensatory capacity, and the risk of THA dislocation, while SS and PT directly regulate functional acetabular anteversion and inclination, with larger PI correlating with greater PT variation and a narrower patient‐specific safe zone. To explore the dynamic variation patterns and coordinated relationships of these pelvic characteristic angles during STS movement, we conduct a laboratory‐based exploratory biomechanical investigation involving healthy volunteers, as shown in Figure 1. The study design includes recruiting eligible participants, acquiring dynamic kinematic data of STS movements, performing inverse kinematics (IK) analysis and biomechanical simulation using OpenSim software, processing data to calculate SS, PT, and PI (with a projection correction algorithm to eliminate systematic errors from 3D pelvic movement), and identifying key stationary points of the angles to analyze their temporal characteristics. Classified as Level V evidence (nonclinical, exploratory), this study does not report clinical outcomes but aims to generate hypotheses for future clinical research on pelvic biomechanics and related movement disorders.

### 2.1 Participants

A total of 10 healthy participants (male:female = 1:1; 19–24 years old) were recruited for this study; all of them signed an informed consent prior to the trials. No participants reported a history of musculoskeletal disorders, neurological diseases, or lower limb injuries that could affect movement function. This study has been reviewed and approved by the Ethics Committee of Peking University Third Hospital (approval number: M20260379). All experimental procedures comply with the ethical guidelines of the Helsinki Declaration, and written informed consent was obtained from all human subjects prior to the commencement of the experiment.

### 2.2 Motion Capture Experiment

Dynamic kinematic data during the STS movement are acquired using the NOKOV motion capture system (Duliang Technology Co. Ltd., China). Eight high‐speed infrared cameras are mounted around the experimental area, forming a capture volume of 3 m × 3 m × 2 m. Marker trajectories are continuously tracked using XingYing software (Version 3.0, Duliang Technology Co. Ltd.), generating time‐series 3D coordinate data. This process establishes a global coordinate system and synchronizes the spatial positions of all cameras to ensure measurement accuracy. The system's spatial resolution is verified to be submillimeter‐level (±0.1 mm).

**FIGURE 2:**
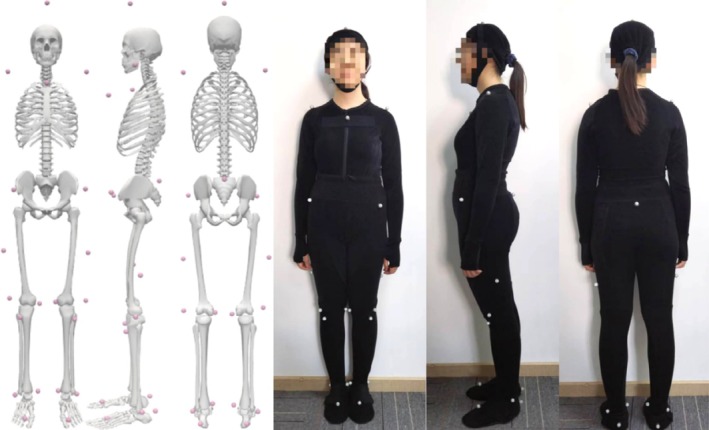
The positions of the markers (based on the Helen Hospital markerset). Twenty‐three marker points are used to capture human body movements in the STS cycle.

Reflective marker spheres (diameter = 14 mm) are affixed to anatomical bony landmarks of the lower extremities and pelvis. Based on the Helen Hospital Marker Set [22], a simplified configuration is adopted to focus on lower limb kinematics, resulting in a total of 23 markers (Figure 2). To ensure marker stability during dynamic movements, all markers are secured via hook‐and‐loop fasteners on a customized, high‐elasticity motion capture suit. This approach minimizes skin motion artifact and maintains consistent marker placement relative to the underlying skeletal segments.

Subjects were instructed to sit on a standard chair (seat height = 45 cm) with feet flat on the ground, arms crossed over the chest. After a 30‐s rest period, subjects performed three consecutive STS movements at a self‐selected, comfortable speed. The middle trial is selected for analysis to avoid initial adaptation and fatigue effects.

### 2.3 Biodynamics Simulation

OpenSim (Version 4.4, Stanford University, USA) is an open‐source biomechanical simulation platform that enables non‐invasive modeling and analysis of human movement [23, 24]. The platform is used for IK solution and extraction of pelvic kinematic parameters, as follows. The standard Gait2352 musculoskeletal model is scaled to match each participant's anthropometric characteristics (height and weight) and static marker positions using the “Scale Model” tool in OpenSim. Segment lengths and masses are adjusted proportionally to minimize kinematic errors. IK is applied to the motion capture data to compute joint angles and segment orientations by minimizing the root‐mean‐square error between experimental marker positions and model‐predicted marker positions. Subsequently, the trajectories of three key points are extracted using the “Point Analyze” function of the software for the subsequent calculation of SS, PT, and PI. These key points included: (1) SM, the midpoint of the upper endplate of the sacrum; (2) SF, a specific point on the surface of the upper endplate of the sacrum; and (3) H, the midpoint of the line connecting the centers of the left and right femoral heads.

The definition of pelvic angles, the local coordinate systems for each segment, and the sign conventions for joint rotations are strictly aligned with the International Society of Biomechanics (ISB) recommendations to ensure reproducibility [25]. The global *Y*‐axis is defined as superior, the *X*‐axis as anterior–posterior, and the *Z*‐axis as medial‐lateral. A brief methodological summary detailing the ISB‐oriented coordinate transformations is provided as Supporting Information S1.

### 2.4 Data Processing and Event Identification

#### 2.4.1 Calculation Method for Pelvic Characteristic Angles

The calculation of pelvic characteristic angles (SS, PT, and PI) is based on the 2D coordinate data (*xy*‐plane) of three key pelvic points (SM, SF, and H) extracted frame‐by‐frame from motion analysis software. First, two core vectors are constructed from the coordinate data: ${\vec{v}}_{12}$ (vector from SM to SF) and ${\vec{v}}_{13}$ (vector from SM to H). For SS, it is calculated as the directional angle of ${\vec{v}}_{12}$, with the formula:

(1) $$\mathrm{SS}=-\deg\left(\arctan2\left({\vec{v}}_{12,y},{\vec{v}}_{12,x}\right)\right)$$

where ${\vec{v}}_{12,x}$ and ${\vec{v}}_{12,y}$ represent the *x* and *y* components of ${\vec{v}}_{12}$, respectively, and the negative sign is introduced to align with the anatomical coordinate system convention.

PT is defined as the directional angle of ${\vec{v}}_{13}$, calculated by

(2) $$\mathrm{PT}=\deg\left(\arctan2\left({\vec{v}}_{13,x},-{\vec{v}}_{13,y}\right)\right)$$

adjusting the component order and sign to conform to pelvic kinematics measurement norms.

All angle results are reshaped into column vectors to match the time series dimension, then saved to an Excel file alongside the time series data, and visualized as time‐series plots to reflect the dynamic changes of SS, PT, and PI over time.

#### 2.4.2 Stationary Point Identification

The STS movement cycle is normalized to a 0%–100% scale. The onset (0%) is defined as the instant the participant's posterior thighs and buttocks initiated seat‐off, which is identified through synchronized visual inspection of the OpenSim IK simulation videos. The completion (100%) is defined as the achievement of a stable, upright standing posture without residual postural sway. To identify the stationary points of pelvic characteristic angles, the raw kinematic trajectories are smoothed using a low‐pass filter (cutoff frequency: 6 Hz) to minimize high‐frequency noise. Stationary points are then defined as the local extrema (maxima for SS and minima for PT) within the smoothed time‐series data. Given the exploratory nature of this study and the small, homogeneous cohort (*n* = 10), this manual‐visual identification approach is prioritized to ensure high anatomical fidelity between the simulation‐derived motion and the corresponding kinematic curves, providing a reliable basis for subsequent biomechanical analysis.

#### 2.4.3 Projection Correction Algorithm

**FIGURE 3:**
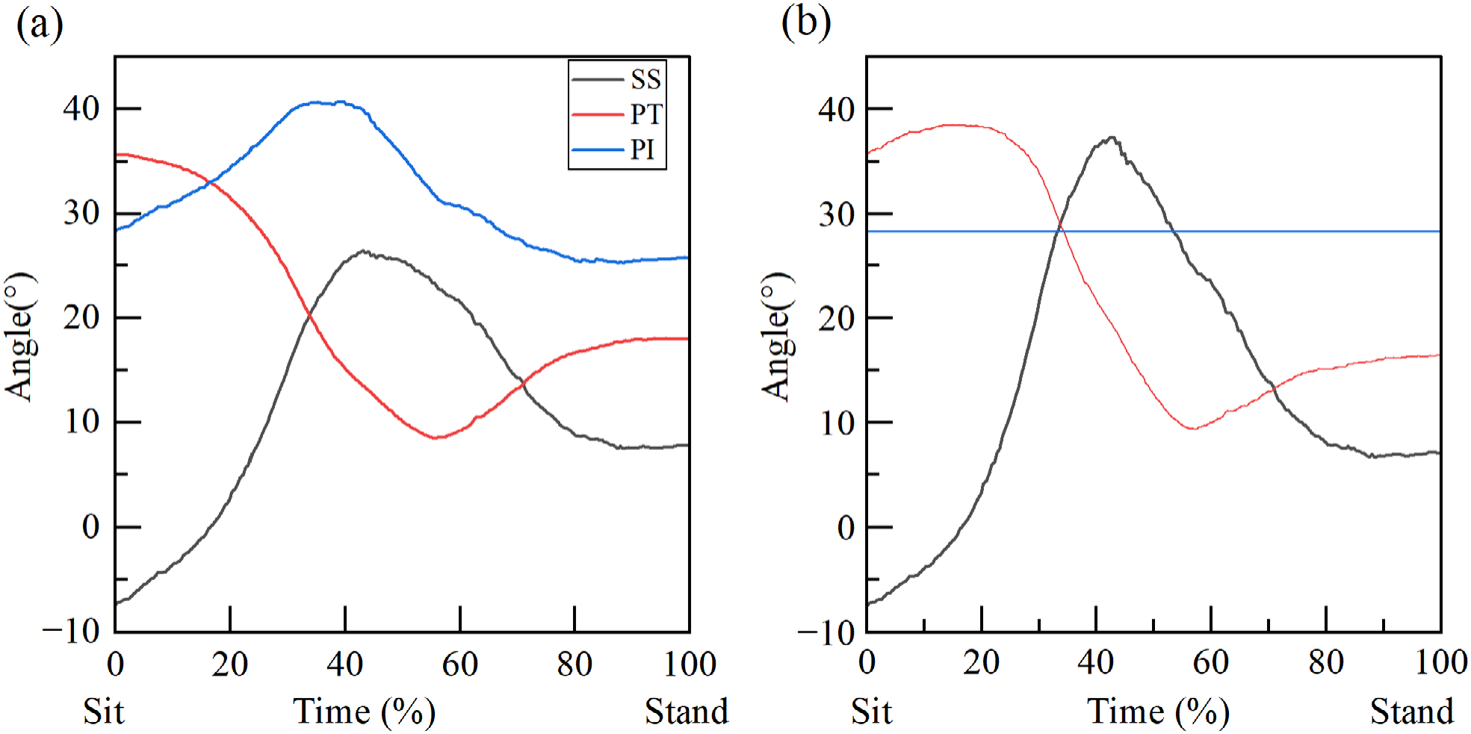
Comparison chart before and after applying the PI correction algorithm: a. The curve of the pelvic characteristic angle change in the original version (with fluctuations). b. The curve of pelvic characteristic angles in the revised version (after correction).

This study employs a global coordinate system for motion capture and data acquisition, and the initial calculation of pelvic characteristic angles is performed based on a fixed global *xy*‐plane (sagittal plane). During STS motion, however, the pelvis does not move within a single plane but undergoes three‐dimensional movements including pelvic obliquity and rotation. As a result, the local coordinate system of the pelvis continuously changes its spatial orientation relative to the fixed global coordinate system of the experiment. This fundamental mismatch leads to the following consequences: the anatomical landmark coordinates extracted from ID results are essentially two‐dimensional projections of the pelvic local coordinate system onto the fixed global sagittal plane; the directly calculated SS, PT, and PI values are projected angles rather than the true anatomical angles under the pelvic local coordinate system; and PI exhibits non‐physiological fluctuations in calculations, which represent systematic errors caused by projection distortion. To obtain true pelvic angles consistent with anatomical definitions, a projection correction algorithm is proposed to inversely correct the measured angles in the global coordinate system to the real angles in the pelvic local coordinate system as shown in Figure 3.

A reference frame **H**‐*xyz* is established with its origin at point **H** and axes parallel to the global coordinate system. As the subject was instructed to sit upright facing the positive *x*‐axis at the initial time, the triangle ΔH−SM−T can be assumed to lie in the *xy*‐plane at *t* = 0, with the true value of the PI angle defined as ${\mathrm{PI}}_{\text{true}}=∠H-\mathrm{SM}-T$. We now assume that ΔHMT undergoes a clockwise rotation $\alpha \;\left(0^{{}^{\circ}}\leq \alpha <360^{{}^{\circ}}\right)$ about the *x*‐axis and a clockwise rotation $\beta \;\left(0^{{}^{\circ}}\leq \beta <360^{{}^{\circ}}\right)$ about the *y*‐axis. At time *t*, the projected points of **SM** and **T** onto the *xy*‐plane are denoted **SM′** and **T′**, respectively. The value of PI computed by our algorithm, which is distorted by projection, is defined as ${\mathrm{PI}}_{\text{measured}}=∠H-{\mathrm{SM}}^{'}-T^{'}$.

The measured angle is obtained by rotating the true angle around the *x*‐axis (angle *α*) and *y*‐axis (angle *β*) and projecting it onto the *xy*‐plane. The cosine relationship between the measured angle and the true angle is as follows:

(3) $$\cos\left(PI_{\text{measured}}\right)=\frac{\cos\left(PI_{\text{true}}\right)\cos\beta -\sin\left(PI_{\text{true}}\right)\sin\alpha \sin\beta }{\sqrt{{\left(\cos\left(PI_{\text{true}}\right)\cos\beta -\sin\left(PI_{\text{true}}\right)\sin\alpha \sin\beta \right)}^{2}+{\left(\sin\left(PI_{\text{true}}\right)\cos\alpha \right)}^{2}}}$$

Given the angle PI is the sum of two sub‐angles SS and PT, this model is also applicable to sub‐angles SS and PT:

(4) $$\cos\left(SS_{\text{measured}}\right)=\frac{\cos\left(SS_{\text{true}}\right)\cos\beta -\sin\left(SS_{\text{true}}\right)\sin\alpha \sin\beta }{\sqrt{{\left(\cos\left(SS_{\text{true}}\right)\cos\beta -\sin\left(SS_{\text{true}}\right)\sin\alpha \sin\beta \right)}^{2}+{\left(\sin\left(SS_{\text{true}}\right)\cos\alpha \right)}^{2}}}$$

(5) $$\cos\left(PT_{\text{measured}}\right)=\frac{\cos\left(PT_{\text{true}}\right)\cos\beta -\sin\left(PT_{\text{true}}\right)\sin\alpha \sin\beta }{\sqrt{{\left(\cos\left(PT_{\text{true}}\right)\cos\beta -\sin\left(PT_{\text{true}}\right)\sin\alpha \sin\beta \right)}^{2}+{\left(\sin\left(PT_{\text{true}}\right)\cos\alpha \right)}^{2}}}$$

where $SS_{\text{true}}=SS_{\text{corrected}},PT_{\text{true}}=PT_{\text{corrected}}$.

Since the rotation angles *α* and *β* cannot be uniquely determined, a linear proportional correction $k=\frac{PI_{\text{true}}}{PI_{\text{measured}}}$ based on the invariance of distortion ratio is adopted in engineering.

Corrected values are calculated as follows:

(6) $$SS_{\text{corrected}}=SS_{\text{measured}}\times \frac{PI_{\text{true}}}{PI_{\text{measured}}}$$

(7) $$PT_{\text{corrected}}=PT_{\text{measured}}\times \frac{PI_{\text{true}}}{PI_{\text{measured}}}$$

## 3 Results

**FIGURE 4:**
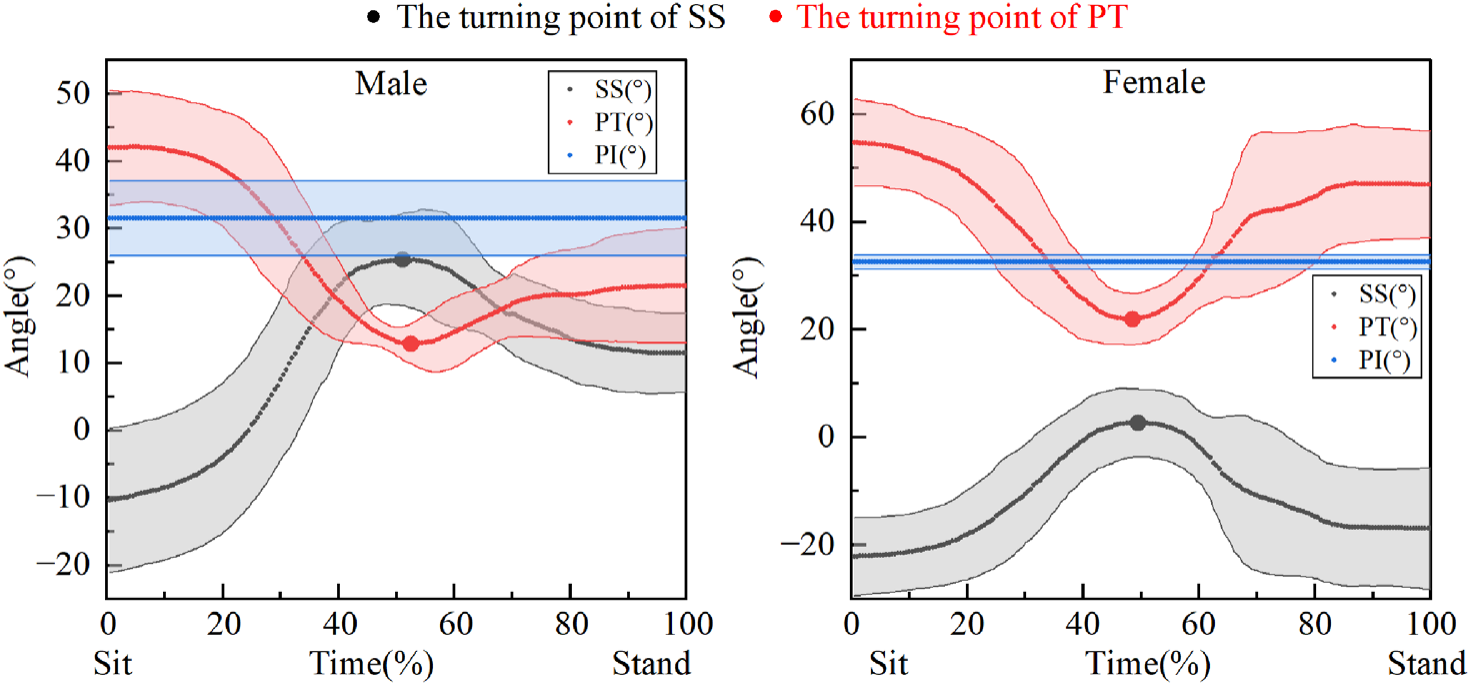
The variation curves of pelvic characteristic angles during the STS movement cycle. The average pelvic characteristic angles of 10 participants (male:female = 1:1) are plotted in the figure. The horizontal axis represents the normalized time, and the vertical axis represents the angle change. The three curves in the figure correspond to SS, PI, and PT angles, respectively.

A total of 60 STS trials are collected from 10 participants (each was tested six times). Figure 4 illustrates a representative STS motion trajectory obtained from OpenSim simulation. Analysis of the kinematic data reveals coordinated and dynamic changes in the pelvic angles (SS, PT, and PI) throughout the movement. Furthermore, notable differences in the ROM are observed between male and female participants.

As anticipated by its anatomical definition as the geometrical sum of SS and PT, the PI angle remains relatively constant [26]. In contrast, the SS and PT angles exhibit substantial, reciprocal changes during the rising phase [27]. Specifically, the transition from sitting to standing demonstrates a dynamic, two‐phase pelvic motion pattern. In the first, or propulsion phase, a rapid decrease in the PT angle is synchronized with an increase in the SS angle. This coordinated action drives the pelvis from a posteriorly tilted position to an anteriorly tilted one, providing the necessary momentum for lifting the center of mass (COM). In the subsequent stabilization phase, a slight reversal is observed, characterized by a small increase in PT and a corresponding decrease in SS. This adjustment signifies the torso recovering from the forward inertia to achieve a stable, upright stance. This pattern indicates that the STS task is not a simple anterior pelvic rotation but a complex motor strategy requiring fine‐tuned coordination between the pelvis and torso [24, 28].

Notably, the temporal profiles of the SS and PT angles are not linear. The angular changes in both the propulsion and stabilization phases are characterized by an initial acceleration followed by deceleration. This “accelerate‐decelerate” feature suggests that the movement is not performed at a constant velocity but relies on active acceleration and controlled deceleration by the neuromuscular system to ensure a smooth, efficient, and stable motion [29].

To further quantify the temporal distribution and amplitude characteristics of the stationary points in pelvic angles, Figure 4 presents the amplitude‐time profiles of ΔSS and ΔPT, where the vertical axis represents the relative angle increment of the stationary points compared to the sitting posture as follows:

(8) $$\Delta {\mathrm{SS}}_{\mathrm{ts}}=\left|{\mathrm{SS}}_{\text{stationary point}}-{\mathrm{SS}}_{\mathrm{sit}}\right|$$

(9) $$\Delta {\mathrm{PT}}_{\mathrm{ts}}=\left|{\mathrm{PT}}_{\text{stationary point}}-{\mathrm{PT}}_{\mathrm{sit}}\right|$$

(10) $$\Delta {\mathrm{SS}}_{\mathrm{ss}}=\left|{\mathrm{SS}}_{\text{stand}}-{\mathrm{SS}}_{\mathrm{sit}}\right|$$

(11) $$\Delta {\mathrm{PT}}_{\mathrm{ss}}=\left|{\mathrm{PT}}_{\text{stand}}-{\mathrm{PT}}_{\mathrm{sit}}\right|$$

and the horizontal axis denotes the normalized time of stationary point occurrence. The time normalization is performed using the formula as follows:

(12) $$t_{\text{normal}}=\frac{t_{\text{real}}-T_{\mathrm{sit}}}{T_{\text{stand}}-T_{\mathrm{sit}}}\times 100\%$$

where *T*_sit_ is the time at the start of the STS movement (sitting posture) and *T*_stand_ is the time at the completion of stable standing, ensuring the entire movement cycle is scaled to a 0%–100% time window for standardized comparison across participants.

**FIGURE 5:**
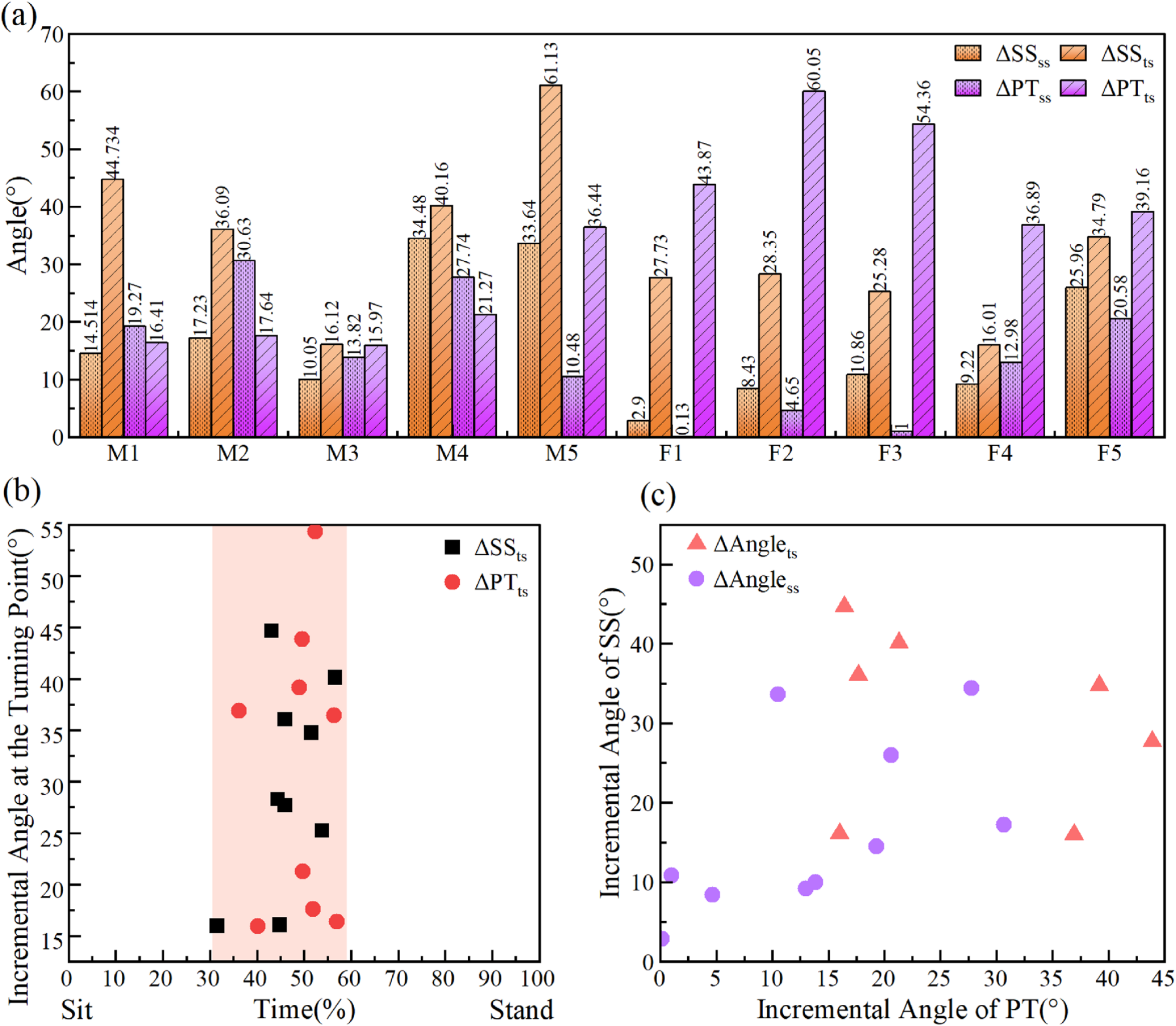
The amplitude‐time characteristics of the stationary points. (a) The variation ranges of the SS and PT angles among 10 volunteers. (b) Relationship between the normalized time corresponding to stationary points and the increments of angles. (c) Comparison of angular differences calculated using stationary points with those measured in sitting and standing positions, where ΔAngle_ts_ refers to ΔSS_ts_ and ΔPT_ts_, and ΔAngle_ss_ refers to ΔSS_ss_ and ΔPT_ss_.

As illustrated in Figure 5, the stationary points of both SS and PT are concentrated within a normalized time interval, which corresponds to the mid‐phase of the STS movement (propulsion phase) identified earlier. At the 95% confidence level, the true mean of the normalized timing corresponding to stationary points for ΔPT is estimated to lie within 44.48%–53.19%, while that for ΔSS falls within 42.00%–52.73%. Specifically, the ΔPT values at the stationary points show a distinct negative increment relative to PT_sit_, reflecting the sharp decline of PT to its trough during this period; in contrast, ΔSS exhibits a positive increment relative to SS_sit_, indicating the synchronous elevation of SS to its peak. Meanwhile, the amplitude discrepancies in their relative increments visually illustrate the magnitude of pelvic posture adjustment during this critical movement phase. This amplitude‐time characteristic provides direct quantitative evidence for the nonlinear dynamic changes of pelvic angles, highlighting that the 42.00%–53.19% normalized time interval is the core phase of pelvic posture transition during STS.

**FIGURE 6:**
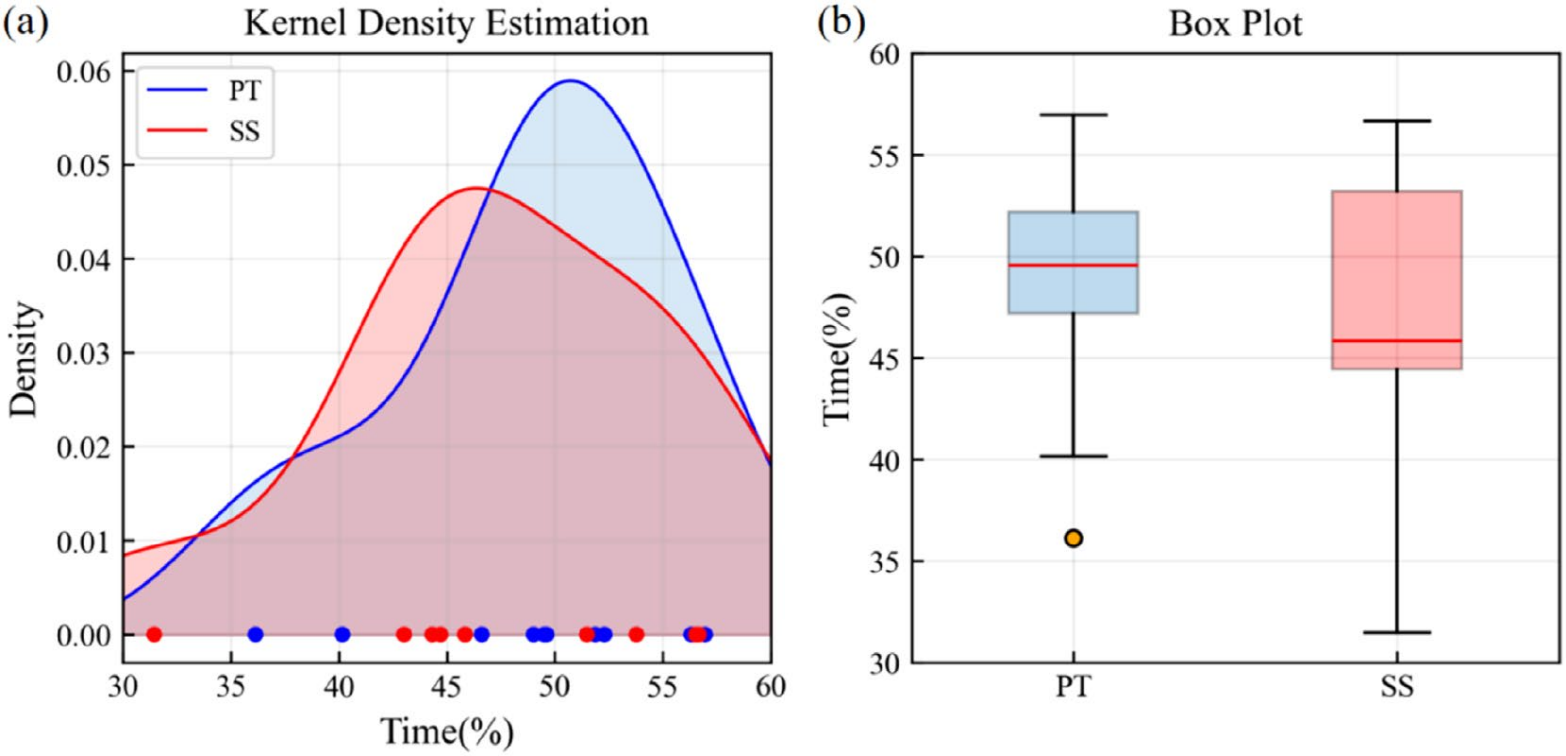
Kernel density estimation and box plot of PT and SS groups. (a) Non‐parametric kernel density estimation curves of the normalized turning times of the PT (blue) and SS (red) angles, with data points marked on the *x*‐axis. (b) Box plots comparing the distribution, median, and dispersion of the two groups, with outliers marked as circles.

As shown in Figure 6, the kernel density estimation and box plot analysis further quantify the robustness and inter‐individual variability of these stationary points. Both PT and SS turning times exhibit unimodal density profiles, with the majority of samples clustered around their respective central tendencies, indicating a consistent temporal pattern of pelvic posture transition. The box plots reveal that the interquartile ranges (IQRs) for PT and SS are relatively narrow, with median values of 49.7% and 45.8%, respectively, demonstrating high robustness of the stationary point timing against individual variations. However, subtle differences in dispersion are observed: the SS group shows a slightly wider IQR and a lower extreme value (31.4%), while the PT group presents a mild outlier at 36.1%, reflecting inherent inter‐individual differences in the initiation and progression of pelvic adjustment during the propulsion phase. These statistical characteristics confirm that while the mid‐term time window serves as a universal core phase for pelvic posture transition, individual motor strategies introduce measurable variability in the exact timing of stationary points.

## 4 Discussion

**FIGURE 7:**
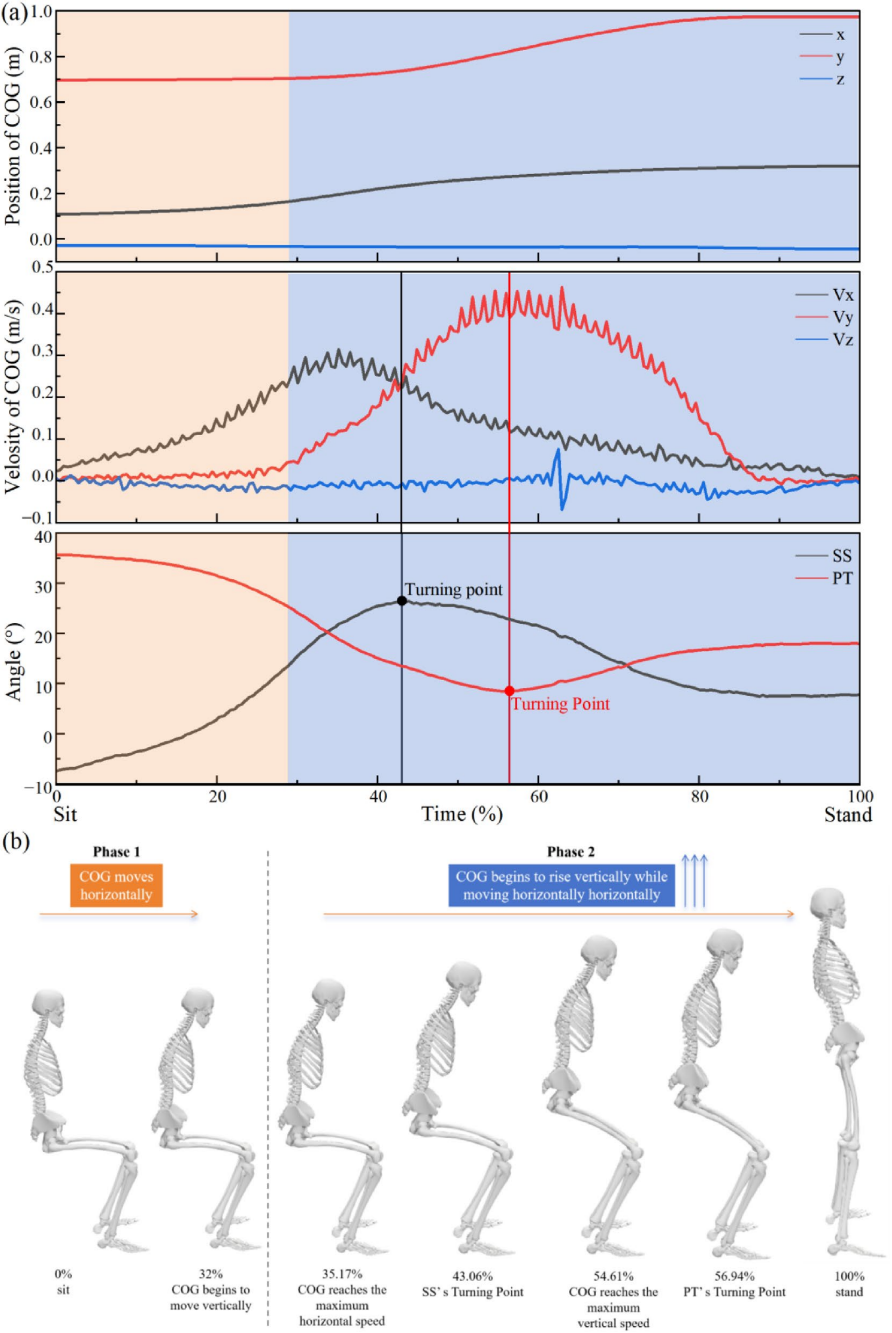
Joint analysis of pelvic characteristic angles and center of gravity. (a) The position‐time curves of the COG, the velocity–time curves of the COG, and the angle–time curves of PT (SS). (b) A schematic diagram of human body movement from sitting to standing.

During the STS movement, the motion can be distinctly divided into two characteristic phases. As shown in Figure 7, a significant synergistic regulation exists between the kinematic features and pelvic angle adjustments, which is clearly reflected in the temporal changes of the COM in three‐dimensional motion and the sagittal pelvic characteristic angles (SS and PT).

The first phase is the pre‐seat‐off phase (approximately 0%–32%), during which the COM displacement is dominated by horizontal movement: the *y*‐axis (vertical) position remains largely stable, while the *x*‐axis (anterior–posterior) position increases rapidly. Concurrently, the *x*‐axis velocity (*V*_*x*_) gradually rises, whereas the *y*‐axis velocity (*V*_*y*_) remains near zero. This pattern reflects the forward horizontal transfer of the COM away from the seat, establishing a biomechanically preparatory posture for the subsequent vertical lift.

In the second phase (approximately 32%–100%), the COM exhibits a composite three‐dimensional motion: the *x*‐axis displacement stabilizes after a minor adjustment, while the *y*‐axis displacement increases rapidly by 0.3 m. In terms of velocity, *V*_*x*_ decreases to zero after a brief acceleration, and *V*_*y*_ rises rapidly to a peak before declining sharply. This dynamic reflects the COM's vertical elevation accompanied by a slight anterior adjustment, accomplishing the spatial transition from sitting to standing. Notably, the peak *V*_*y*_ coincides with the trough of the PT angle (approximately 57%); the eventual stabilization of both the SS and PT angles marks the establishment of a balanced biomechanical alignment among the spine, pelvis, and lower limbs in the standing posture [30, 31].

These results confirm that dynamic sagittal pelvic alignment (reflected in the temporal variation of SS/PT) serves as a core regulatory mechanism for COM control during STS: the first phase employs active anterior PT to shift the COM over the base of support, laying the foundation for the mechanical initiation of STS motion; the second phase relies on the recovery of pelvic posture to achieve standing balance. The coordination between the two phases highlights the postural regulatory role of the lumbopelvic complex during STS. The relationship between kinematics and pelvic posture provides critical biomechanical insights for optimizing movement efficiency during STS transitions and for quantitatively assessing abnormal motor patterns.

The ${\mathrm{PI}}_{\text{measured}}$ exhibited certain fluctuations, which contradicted the anatomical definition of PI as a constant morphological parameter. To avoid conceptual ambiguity, this value should be interpreted as a model‐based predictive estimate rather than an actual change in the skeletal structure. This discrepancy primarily arises from the fact that the sagittal plane used for angle calculation is defined as a fixed plane based on the initial body orientation, whereas the functional sagittal plane continuously changes its orientation during dynamic movements. During the STS task, the pelvis exhibits non‐zero obliquity and rotation, and the characteristic angles of the bilateral hip, knee, and ankle joints show significant asymmetry. These out‐of‐plane movements cause displacements of the 3D anatomical landmarks relative to the fixed projection plane, thereby introducing minor mathematical fluctuations in the calculated PI. To address this issue, we developed a projection correction algorithm, which uses the discrepancy between PI_measured_ and ${\mathrm{PI}}_{\text{true}}$ to correct the fluctuations in SS and PT angles, yielding more accurate measurements of the key pelvic angles. In addition, skin motion artifacts (marker displacement) and the inherent geometric approximation of the scaled OpenSim musculoskeletal model [32] may also introduce minor measurement errors.

**FIGURE 8:**
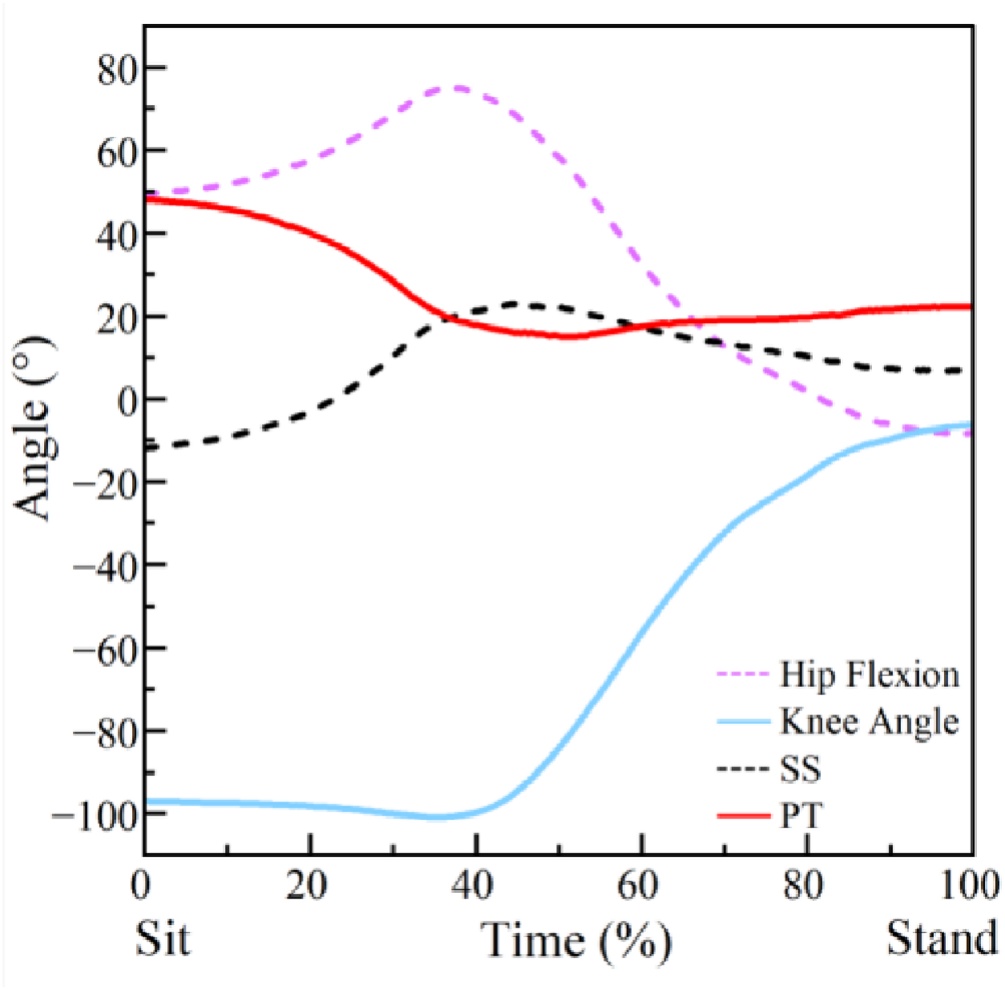
The rule between the characteristic angles of the pelvis and the angle changes of the knee and hip joints.

By integrating the rotational angle characteristics of the hip and knee joints in the sagittal plane (Figure 8), an analysis of the dynamic variation mechanisms of the PT angle and SS angle reveals that: the reduction in PT angle and increase in SS angle are primarily driven by the trunk‐thigh folding movement, that is, hip joint flexion; in contrast, the increase in PT angle and decrease in SS angle result from the synergistic effect of hip joint extension and knee joint extension [33, 34].

It is also noteworthy that anomalous instances where PT exceeds PI occur at specific time points. The underlying causes can be attributed to the following two aspects: Firstly, the seat height was set at 45 cm, which led some subjects to lean their upper bodies backward while seated, resulting in a negative SS angle (SS < 0). These subjects restored the normal biomechanical state where PT < PI after standing upright. Secondly, certain subjects (more frequently observed in female participants) exhibited SS < 0 in both sitting and standing postures, indicating a persistent posterior PT [35, 36, 37].

Previous patient‐specific safe zone algorithms by Tang et al. proposed acetabular and stem positioning guidelines based solely on static PT measurements in sitting and standing postures [38]. The present dynamic biomechanical analysis in young healthy individuals demonstrates that PT and SS exhibit distinct stationary points during the mid‐phase of STS cycle (42.00%–53.19%), where PT reaches a more extreme trough than either static sitting or standing positions. This biomechanical observation suggests that safe zones defined by two static postures may not capture the full range of functionally relevant pelvic orientations, which could theoretically leave patients vulnerable to prosthetic impingement or instability during dynamic daily movements.

High‐level evidence from a recent meta‐analysis further supports the physiological relevance of these dynamic findings by confirming that PT remains remarkably stable even after major pelvic reorientation procedures such as periacetabular osteotomy [39]. The conserved nature of PT across both surgical intervention and physiological movement strengthens the validity of the observed stationary points as consistent, reproducible features of spinopelvic kinematics rather than transient or incidental variations.

Together, these biomechanical observations support the working hypothesis that dynamic pelvic kinematics during functional tasks such as STS represent an essential, previously overlooked component of patient‐specific safe zone planning. Incorporating the critical stationary points of pelvic orientation identified in this study may hypothetically improve the robustness of THA component positioning beyond what is achievable with static sitting‐standing assessments alone. However, future clinical studies in THA populations are necessary to validate this preliminary biomechanical inference and confirm its translational value.

## 5 Limitations and Directions for Future Research

Despite its contributions, this study has several inherent limitations. First, the sample size is relatively small (10 healthy young adults), and the participants are limited to a narrow age range (19–24 years) without including populations with pathophysiological conditions. As demonstrated by Ozyurek et al. [40], persons with transtibial amputation exhibit altered movement strategies during STS, indicating that pelvic dynamic patterns may vary significantly across different populations—this heterogeneity is not addressed in the current study. Second, the research focuses solely on kinematic analysis of pelvic angles, lacking integration with kinetic data (e.g., ground reaction forces) and muscle activation patterns. Davidson et al. [41] highlighted that muscle activation and coactivation are critical for STS performance in patients undergoing total knee arthroplasty, suggesting that omitting these parameters limits a comprehensive understanding of movement mechanisms. Third, the motion capture relied on marker‐based technology, which may introduce errors due to skin motion artifacts, and the scaled OpenSim model cannot fully replicate individual anatomical differences. Although Lee et al. [42] validated the reliability of multi‐view image‐based motion analysis systems, this study did not adopt such advanced technologies to optimize measurement accuracy. Fourth, the STS task was conducted with a fixed seat height (45 cm), and the influence of variable seat heights or assistive devices on pelvic dynamics is not explored. Finally, the study did not establish a direct link between dynamic pelvic parameters and clinical outcomes (e.g., prosthetic impingement in THA patients), limiting the translation of its findings to clinical practice.

Future studies will require larger sample sizes with a minimum of 15 participants per group, as well as the inclusion of age‐appropriate cohorts including individuals over 60 years of age and those with degenerative spinal disorders such as spinal stenosis or degenerative scoliosis, to improve generalizability to typical THA populations. Second, comparative investigations should be undertaken across three rigorously stratified subgroups: healthy controls, uncomplicated THA patients, and THA patients with imaging‐confirmed prosthetic impingement, documented dislocation, or revision surgery, so that causal associations between dynamic pelvic kinematics and adverse clinical outcomes may be established. The influence of variable seat heights within a clinically relevant range (40–50 cm) on pelvic dynamics should also be evaluated to replicate real‐world functional conditions. Finally, implant positioning stability should be simulated under dynamic STS conditions using projection‐corrected PT values, with patient‐specific algorithms such as those described by Tang et al. employed as a reference framework, to develop quantifiable biomechanical parameters for a dynamic acetabular safe zone including PT velocity thresholds at stationary points, and validate the predictive performance of this model in longitudinal clinical studies.

## 6 Conclusion

This study provides novel biomechanical insights into the nonlinear dynamics of pelvic characteristic angles and the coordinated regulation between pelvic posture and COM control during the STS movement. The acetabular safe zone currently fails to capture dynamic pelvic variations, and the critical stationary points of pelvic angles observed during the mid‐STS phase (42.00%–53.19%) may theoretically support preoperative planning and postoperative rehabilitation for THA patients. Given the limited cohort of young healthy individuals, all potential clinical implications for THA and orthopedic rehabilitation remain hypothetical. Further investigations in larger and more diverse cohorts are warranted to translate these biomechanical findings into clinical practice.

## Author Contributions

**Haoyue Xin:** investigation, methodology, visualization. **Chen‐Xu Liu:** writing – review and editing, conceptualization, methodology, software, supervision, investigation. **Minwei Zhao:** supervision, project administration, funding acquisition, conceptualization, writing – review and editing. **Zhanli Liu:** funding acquisition, supervision, project administration, conceptualization. **Hua Tian:** supervision, resources. **Houwen Zheng:** writing – original draft, writing – review and editing, software, methodology, data curation, investigation, validation, formal analysis, visualization.

## Funding

This work was supported by the National Natural Science Foundation of China, 82272576, 12525208, and 12402103.

## Disclosure

All authors listed meet the authorship criteria according to the latest guidelines of the International Committee of Medical Journal Editors, and all authors are in agreement with the manuscript.

## Ethics Statement

All participating healthy volunteers provided written informed consent prior to enrollment, including consent to participate in the study and publish de‐identified data. The study was conducted in compliance with the Declaration of Helsinki.

## Conflicts of Interest

The authors declare no conflicts of interest.

## Supporting information


Supporting Information: S1.

## Data Availability

The data that support the findings of this study are available on request from the corresponding author. The data are not publicly available due to privacy or ethical restrictions.
