# Supplementary material for: Temporal Evolution and Stationary Point Mechanisms of Pelvic Characteristic Angles Throughout the Sit‐to‐Stand Cycle
Source: Orthop Surg. 2026 Jul 8;18(8):1688–99. doi: 10.1111/os.70366 (PMC13398672; doi:10.1111/os.70366)
Supplement: Supplementary file 1 — Supporting Information: S1. [file OS-18-1688-s001.docx]

**Supplementary Material 1: Rotation Matrix Derivation for Angle Projection Correction**

To derive the relationship between the true three‑dimensional pelvic anatomical angles (true PI, SS, PT) and their two‑dimensional projected measurements under pelvic rotation, we first establish a right‑handed coordinate system with the origin located at the anatomical landmark SM, where the initial vectors defining each angle lie in the transverse *xy*‑plane. The pelvis undergoes successive clockwise rigid rotations: a rotation *α* about the *x*‑axis and a rotation *β* about the *y*‑axis, described by the combined three‑dimensional rotation matrix *R*(*α,β*)=*R_y_*(*β*)*R_x_*(*α*), where $R_{x}(\alpha)=\left[ \begin{matrix} 1 & 0 & 0 \\ 0 & \cos\alpha& \sin\alpha\\ 0 & -sin\alpha& \cos\alpha\end{matrix} \right]$ and $R_{y}(\beta)=\left[ \begin{matrix} \cos\beta& 0 & -sin\beta\\ 0 & 1 & 0 \\ sin\beta& 0 & \cos\beta\end{matrix} \right]$.

By applying this rotation matrix to the unit vectors representing the true anatomical angle in the initial plane, we obtain rotated spatial vectors, which are then orthogonally projected onto the *xy*‑plane by discarding the *z*‑component to simulate planar radiographic measurement. Using the vector dot‑product definition of the included angle, the cosine of the projected measured angle is expressed as the ratio of the dot product of the two projected vectors to the product of their Euclidean norms. Substituting the rotated vector components and simplifying the trigonometric expression under the assumption of small‑to‑moderate rotation angles where cos*β*>0, we derive the unified projection correction formula

$\cos\text{(}\theta_{\text{measured}}\text{)=}\frac{\cos\text{(}\theta_{\text{true}}\text{)}\cos\text{β}\text{-}\sin\text{(}\theta_{\text{true}}\text{)}\sin\text{α}\sin\text{β}}{\sqrt{\text{(}\cos\text{(}\theta_{\text{true}}\text{)}\cos\text{β}\text{-}\sin\text{(}\theta_{\text{true}}\text{)}\sin\text{α}\sin\text{β}\text{)}^{\text{2}}\text{+(}\sin\text{(}\theta_{\text{true}}\text{)}\cos\text{α}\text{)}^{\text{2}}}}$ (S1)

applicable to PI, SS, and PT. Given the non‑uniqueness of reconstructing 3D rotation angles from 2D projections alone, a linear proportional correction based on the ratio of the true angle to the measured angle is adopted for practical clinical and biomechanical applications, enabling consistent correction of projection‑distorted angle measurements under arbitrary sagittal and coronal pelvic rotations.
